# Supplementary material for: Progressive Immunodeficiency with Gradual Depletion of B and CD4+ T Cells in Immunodeficiency, Centromeric Instability and Facial Anomalies Syndrome 2 (ICF2)
Source: Diseases. 2019 Apr 4;7(2):34. doi: 10.3390/diseases7020034 (PMC6631482; doi:10.3390/diseases7020034)
Supplement: Supplementary file 1 [file diseases-07-00034-s001.zip › diseases-467535-supplementary/diseases-467535-Table S1.docx]

**Table S1**

Genotype, phenotype, immunological findings, treatment and outcome of all published ICF2 patients.

| **Nr.** | **Mutation** | **Protein** | **Sex** | **Age at time of last reporting** | **Cause**  **of**  **death** | **Ethnicity** | **facial anomalies** | **motor development** | **intellectual development** | **infections** | **immunological findings** | **Other** | **Therapy** | **References** |
| --- | --- | --- | --- | --- | --- | --- | --- | --- | --- | --- | --- | --- | --- | --- |
| **1** | c.[47C>G];[47C>G] | p.[(Ser16*)];[(Ser16*)] | f | died at the age of 13 y | bronchopneumonia | Scottish | flattend nasal bridge, triangular face, upturned nose, frontal bossing sparse hair | normal | talked three words at 25 months | bronchopneumonia, *Pneumocystis jiroveci* pneumonia, Candida | panhypogammaglobulinemia |  |  | de Greef et al. 2011,  Wemaes et al. 2013, |
| **2** | c.[501dup];[501dup] | p.[(Val168Serfs*28)];[(Val168Serfs*28)] | m | 13 y |  | Turkish | present (not specified) |  | delayed | bronchopneumonia, *Pneumocystis jiroveci* | agammaglobulinemia, reduced T cell proliferation to PAH/sIL2 |  |  | de Greef et al. 2011,  Wemaes et al. 2013 |
| **3** | c.[396_397delTA];[396_397delTA] | p.[(His132Glnfs*19)];[(His132Glnfs*19)] | m | 13 y |  | Lebanese | round face, everted lower lips, high-arched palate, small chin retrognathia | walked at the age of 2.5 y | talked at 4 years |  | normal B cell count with no CD19^+^CD27^+^ memory B cells, low IgM, normal T cell count with high naive/memory CD4^+^ T cell ratio, normal NK cell count |  |  | Chouery et al. 2011,  Wemaes et al. 2013 |
| **4** | c.[396_397delTA];[396_397delTA] | p.[(His132Glnfs*19)];[(His132Glnfs*19)] | m | 12 y |  | Lebanese | round face, everted lower lips, high-arched palate, small chin retrognathia | walked at the age of 2.5 y | talked at 4 years |  | normal B cell count with no CD19^+^CD27^+^ memory B cells, low IgG and IgM, normal T cell count with high naive/memory CD4^+^ T cell ratio, normal NK cell count |  |  | Chouery et al. 2011,  Wemaes et al. 2013 |
| **5** | c.[396_397delTA];[396_397delTA] | p.[(His132Glnfs*19)];[(His132Glnfs*19)] | m | 7 y |  | Lebanese | round face, everted lower lips, high-arched palate, small chin retrognathia | walked at the age of 2.5 y | talked at 4 years |  | normal B cell count with no CD19^+^CD27^+^ memory B cells, low IgG and IgM, normal T cell conut with high naive/memory CD4^+^ T cell ratio, normal NK cell count |  |  | Chouery et al. 2011,  Wemaes et al. 2013 |
| **6** | c.[759C>G];[759C>G] | p.[(Thr253*)];[(Thr253*)] | m |  |  | Turkish | hypertelorism | normal | delay of speech |  | normal B cell count, panhypogammaglobulinemia | hypospadia, brain with distinct bilateral small areas suspicious of focal cortical heterotopy | IVIG | Wemaes et al. 2013,  Kloeckener-Gruissem et al. 2005 |
| **7** | brother of patient 6, born to the same consanguineous parents, therefore same *ZBTB24* variant suspected |  | m | died at 4.5 y | meningoeancephalitis of unknown origin | Turkish | hypertelorism | retarded | delay of speech | several bacterial infections, meningoencephalitis | panhypogammaglobulinemia | hypospadia, umbilical hernia | IVIG | Kloeckener-Gruissem et al. 2005 |
| **8** | c.[833C>G];[1222T>G] | p.[(Ser278*)];[(Cys408Gly)] | m | died at 7 y | Hodgkin’s lymphoma | German | broad nasal bridge, hypertelorism, flat philtrum | walked at the age of 2 y, muscular hypotony of lower extremities | retarded | pyelonephritis, recurrent otitis, pneumonia, gastroenteritis, recur. Impetigo | severe hypogammaglobulinemia, IgM: 0 g/l | Hodgkin lymphoma |  | de Greef et al. 2011,  Wemaes et al. 2013,  Schuetz et al. 2007 |
| **9** | c.[833C>G];[1222T>G] | p.[(Ser278*)];[(Cys408Gly)] | f |  |  | German | broad nasal bridge, hypertelorism, flat philtrum | retarded | retarded | bronchopneumonia | hypogammaglobulinemia |  |  | de Greef et al. 2011,  Wemaes et al. 2013,  Schuetz et al. 2007 |
| **10** | c.[917delA];[ 917delA] | p.[(Asn306llefs*4)];[Asn306llefs*4)] | m |  |  | Turkish | present (not specified) | retarded | retarded | respiratory infections, candida | agammaglobulinemia, reduced T cell proliferation to PAH/sIL2 |  | IVIG | de Greef et al. 2011,  Wemaes et al. 2013 |
| **11** | c.[958C>T];[958C>T] | p.[(Arg320*)];[(Arg320*)] | f |  | pseudomonas sepsis | Turkish | present (not specified) | retarded | retarded | bronchopneumonia,  pseudomonas sepsis | panhypogammaglobulinemia |  |  | de Greef ez al. 2011,  Wemaes et al. 2013 |
| **12** | c.[958C>T];[958C>T] | p.[(Arg320*)];[(Arg320*)] | m | died at 41 y | PML | Japanese | hypertelorism, micrognathia, low set ears |  | delay of speech, IQ 47, learning difficulties | recurrent pneumonias, recurrent sinusitis, JC virus-associated leukoencephalopathy | panhypogammaglobulinemia, low B cell counts with low CD19^+^CD27^+^ memory B cells, no responses to vaccines, low T cell proliferation to mitogens, low NK cell activity |  | IVIG, cytarabine against PML | Nitta et al. 2013,  Kamae et al. 2018 |
| **13** | c.[980_981delGT];[787A>T] | p.[(Cys327Trpfs*54)];[(Lys263*)] | f | 18 y |  | Cape Verdean | epicanthic folds |  | IQ<70, delay of speech, no reading until 16 y | recurrent thrush by candida in 1st year, recurrent ear, nose and throat infections, recurrent bacterial pulmonary infections (*S. pneumoniae*), bronchiectasis and atelectasis, lobotomy, severe mononucleosis | low 27^+^ B cells, low IgM, no vaccine response to pneumococcus/ poliovirus/ diftheria/ tetanus toxoid, no IgM isoheamaglutinins, normal proliferation to PHA and tetanus and low to candida, NK cell count normal |  | IVIG, azithromycin, otrimoxazole | Nitta et al. 2013,  Sterlin et al. 2016 |
| **14** | c.[1148G>A];[1148G>A] | p.[(Cys383Tyr)];[(Cys383Tyr)] | m | died at 7 y | severe viral infection | Japanese | macrocephaly, hypertelorism, epicanthalfolds, midface flatness, low nasal root, long flat philtrum, thick lips (upper lips<lower lips), genu valgum, irregular toes, hypoplastic primary teeth | delayed | normal | refractory diarrhea, recurrent respiratory tract infections, pneumococcemia, skin infection, CMV viremia, persistent EBV infection | low CD27^+^ B cells, low IgM and low IgG2, decreased NK cell activity, no responses to vaccines | hypopituitarism, bilateral hydronephrosis, skin blisters in case of fever, freckles when sun, butterfly erythema | IVIG | Nitta et al. 2013,  Kamae et al. 2018 |
| **15** | c.[1222T>G];[1222T>G] | p.[(Cys408Gly)];[(Cys408Gly)] | m | 8 y |  | Moroccan | broad depressed nasal bridge | walked at 20 months | IQ65, first words at 12 months, delay of speech |  | low IgM and low CD16^+^ NK cell count, reduced T cell proliferation to PAH/sIL2 | multiple café-au-lait spots since birth |  | Cerbone et al. 2012,  Wemaes et al. 2013 |
| **16** | c.[1369C>T];[1369C>T] | p.[(Arg457*)];[(Arg457*)] | m | adult |  | Italian | epicanthus, hypertelorism, flat nasal bridge, low set ears |  | IQ 61 | bronchopneumonia, recurrent bronchiolitis | low IgG an IgA, low CD4+T cells (270/µ), low CD19+ B cells | failure to thrive |  | Greef et al. 2011,  Wemaes et al. 2013 |
| **17** | c.[958C>T];[958C>T] | p.[(Arg320*)];[(Arg320*)] | m | 3 y |  | Turkish | present (not specified) |  |  |  | panhypogammaglobulinemia, reduced T cell proliferation to PAH/sIL2 |  |  | Wemaes et al. 2013 |
| **18** | c.[1222T>G];[1222T>G] | p.[(Cys408Gly)];[(Cys408Gly)] | f | 9 y |  | German | hypertelorism, epicanthal folds, flat nasal bridge, slight ptosis, prominent forehead, large teeth due to fusion of first molars and incisors |  |  | recurrent upper airway infections, pneumonia at 2.5 years (*E. cloacae*), recur. protracted diarrhea (enteropathogenic *E. coli*), a prolonged skin infection *S. pyogenes* | low IgG and IgM, no antibody response to tetanus toxoid and pneumococcal vaccines, lack of isoheamaglutinins, low B and NK cell counts, reduced PAH, anti-CD3 and antigen (Candida, tetanus toxoid and PPD-induced lymphocyte proliferation, low NK cell cytotoxicity | massive hepatosplenomegaly, liver cirrhosis by 9-years of age, interstitial granulomatous nephritis, fingers and toes with clubbing, failure to thrive | SCIg | von Bernuth et al. 2014 |
| **19** | c.[958C>T];[958C>T] | p.[(Arg320*)];[(Arg320*)] | m | 11 y |  | Turkish | hypertelorism, broad nasal bridge, long philtrum, small low-set ears |  | IQ 49 | recurrent respiratory tract infections, protracted CMV infection at the age of 3 years, EBV-induced hemophagocytic lymphohistiocytosis | low IgM and low IgG | café-au-lait spots, sparce hair, transplanted due to hemophagocytic lymphohistiocytosis | HSCT | Harnisch et al. 2016 |
| **20** | c.[917delA];[ 917delA] | p.[(Asn306llefs*4)];[Asn306llefs*4)] | m | 16 y |  | Turkish | hypertelorism, flat nasal bridge, epicanthus, up-turned nose, macroglossia, telecanthus, micrognathia, low-set ears, round face |  | delayed speech development, retarded | resp. infections such as bronchopneumonia, Candida, CMV-Infection, *Pneumocystis jirovecii* pneumonia | panhypogammaglobulinemia, low B cell counts |  | IVIG | van den Boogaard et al. 2017 |
| **21** | c.[917delA];[ 917delA] | p.[(Asn306llefs*4)];[Asn306llefs*4)] | m | 5 y |  | Turkish | hypertelorism, flat nasal bridge, epicanthus, up-turned nose, telecanthus, micrognathia, low-set ears, round face |  | delayed speech development, retarded | resp. Infections such as bronchopneumonia | panhypogammaglobulinemia, low B cell counts |  | IVIG | van den Boogaard et al. 2017 |
| **22** | c.[917delA];[ 917delA] | p.[(Asn306llefs*4)];[Asn306llefs*4)] | m | 16 y |  | Turkish | hypertelorism, flat nasal bridge, up-turned nose, macroglossia, telecanthus, round face |  | delayed speech development, retarded | respiratory infections such as bronchopneumonia, meningitis (*S. pneumoniae*) | panhypogammaglobulinemia, low B cell counts |  | IVIG | van den Boogaard et al. 2017 |
| **23** | c.[909dup];[909dup] | p.[(Lys304*)];[(Lys304*)] | m | 32 y |  |  | hypertelorism, flat nasal bridge, epicanthus, telecanthus, micrognathia |  | delayed speech development | otitis, resp. infections such as bronchopneumonia | low IgM and IgG | seizures | IVIG | van den Boogaard et al. 2017 |
| **24** | homozygous deletion on chr. 6 including *ZBTB24* |  | m | 22 y |  |  | hypertelorism, flat nasal bridge, epicanthus, micrognathia, low-set ears |  | delayed speech development, retarded | resp. infections such as bronchopneumonia, meningitis (*S. pneumoniae*) | panhypogammaglobulinemia, low B cell counts | seizures | IVIG | van den Boogaard et al. 2017 |
| **25** | c.[1396C>T];[1396C>T] | p.[(Arg457*)];[(Arg457*)] | f | 12 y |  | Japanese | hypertelorism |  | IQ 75 | recurrent pneumonia | low IgG and IgA, low CD27^+^ memory B cells |  | IVIG, TMP/SMX, antifungal drug (not stated) | Kamae et al. 2018 |
| **26** | c.[1108_1109dup];[1108_1109dup] | p.[(Ser370Lysfs*12)];[(Ser370Lysfs*12)] | m | 28 y |  | Japanese | hypertelorism, broad flat nasal bridge |  | normal work without difficulty | recurrent pneumonia, sinusitis | panhypogammaglobulinemia, low IgM^+^CD27^+^ memory B cells, low NK cell count |  | IVIG, TMP/SMX | Kamae et al. 2018 |
| **27** | c.[175A>G];[175A>G] | p.[(Ser59Gly)];[(Ser59Gly)] | m | 24 y |  | Italian | dolichocephaly, high forehead, hypertelorism/telecanthus |  |  | EBV-viremia, recurrent stomatitis and bronchopneumonias | low IgG and IgM, low CD19^+^ cells, relative increase in increased CD8^+^ T cell count | cryptorchidism, chronic autoimmune cholangitis, EBV-related Hodgkin-lymphoma at the age of 27 y | IVIG | Licciardi et al. 2019 |
| **28 (P1)** | c.[1222T>G];[1222T>G] | p.[(Cys408Gly)];[(Cys408Gly)] | m | 28 y |  | German | broad flat nasal bridge, micrognathia | normal | attention deficit hyperactivity disorder, treatment with methylphenidate till the 15^th^ year of life, otherwise normal, work without difficulty | sepsis after birth,  three pneumonias (*H. influenzae*),  recurrent upper respiratory tract infections (infections, otitis media, sinusitis) | low IgG and IgM, low CD19^+^ cells, low NK cells and function | atopic dermatitis | SCIg, TMP/SMX, fluconazole | This work |
| **29 (P2)** | c.[1222T>G];[1222T>G] | p.[(Cys408Gly)];[(Cys408Gly)] | f | 24 y |  | German | broad flat nasal bridge | normal | selective mutism in early childhood, otherwise normal, work without difficulty | atypical mycobacteriosis,  recurrent shingles, prolonged fever after vaccination with MMR | low IgM and IgG4, low CD19^+^CD27^+^ cells, low NK cells and function | atopic dermatitis, scoliosis | No treatment | This work |
| **30 (P3)** | c.[1222T>G];[1222T>G] | p.[(Cys408Gly)];[(Cys408Gly)] | f | 24 y |  | German | broad flat nasal bridge | normal | selective mutism in early childhood, otherwise normal, work without difficulty | none | low IgM and IgG4, low CD19^+^CD27^+^ cells, low NK cells and function | atopic dermatitis | No treatment | This work |
